# Limitations of Ferroptosis Inhibitors on the Doxorubicin-Induced Cardiotoxicity

**DOI:** 10.3390/antiox15010027

**Published:** 2025-12-24

**Authors:** Yun-Ji Cha, Sae-Bom Jeon, Chan Joo Lee, Hyeong-Jin Kim, Sun-Ho Lee, Hyoeun Kim, So Hee Park, Elaine Zhelan Chen, Jong Woo Kim, Sahng Wook Park, Chulan Kwon, Boyoung Joung, Eun-Woo Lee, Seunghyun Lee

**Affiliations:** 1 Department of Biochemistry and Molecular Biology, Institute of Genetic Science, Yonsei University College of Medicine, 50-1 Yonsei-ro, Seodaemun-gu, Seoul 03722, Republic of Korea; ji9602@yuhs.ac (Y.-J.C.); shlee0626@yuhs.ac (S.-H.L.); khe7813@yuhs.ac (H.K.); swpark64@yuhs.ac (S.W.P.); 2 Department of Biochemistry and Molecular Biology, Ajou University School of Medicine, Suwon 16499, Republic of Korea; jsb02257@ajou.ac.kr; 3 Inflamm-Aging Translational Research Center, Ajou University Medical Center, Suwon 16499, Republic of Korea; 4 Division of Cardiology, Severance Cardiovascular Hospital, Cardiovascular Research Institute, Yonsei University College of Medicine, 50-1 Yonsei-ro, Seodaemun-gu, Seoul 03722, Republic of Korea; zanzu@yuhs.ac; 5 Division of Cardiology, Department of Medicine, Johns Hopkins University School of Medicine, Baltimore, MD 21205, USA; zchen119@jhmi.edu (E.Z.C.); ckwon13@jhmi.edu (C.K.); 6 Metabolic Regulation Research Center, Korea Research Institute of Bioscience and Biotechnology (KRIBB), Daejeon 34134, Republic of Korea; wmf5885@kribb.re.kr; 7 School of Pharmacy, Sungkyunkwan University, Suwon 16419, Republic of Korea; 8 Department of Functional Genomics, University of Science and Technology (UST), Daejeon 34113, Republic of Korea

**Keywords:** ferroptosis, doxorubicin-induced cardiotoxicity, human iPSC-derived cardiomyocytes

## Abstract

Doxorubicin is an anthracycline anticancer drug commonly used to treat lymphoma and breast cancer. Its major side effect is cardiotoxicity, which occurs by damaging cardiomyocytes. The mechanisms of doxorubicin-induced cardiotoxicity remain unclear; however, recent studies suggest that ferroptosis, an iron-dependent form of lipid peroxidation-mediated cell death, may play a key role. In this study, we investigated the role of ferroptosis in doxorubicin-induced cardiotoxicity using ferroptosis-specific inhibitors (ferrostatin-1 and liproxstatin-1). In both H9c2 cardiomyocyte cell lines and human induced pluripotent stem cell-derived cardiomyocytes, ferrostatin-1 and liproxstatin-1 rescued cell death induced by RSL3, a ferroptosis inducer, but failed to prevent doxorubicin-induced cell death. Additionally, the ferroptosis inhibitors did not restore the electrophysiological function of cardiomyocytes, measured using a multi-electrode array system, and instead slightly accelerated cardiomyocyte beating. Finally, doxorubicin-injected mice treated with ferroptosis inhibitors exhibited significantly reduced survival and increased levels of N-terminal pro B-type natriuretic peptide, a biomarker of heart failure. These findings suggest that inhibiting ferroptosis alone is insufficient to mitigate doxorubicin-induced cardiotoxicity.

## 1. Introduction

Doxorubicin (DOX) is a potent chemotherapeutic agent widely used to treat various types of cancer owing to its high efficacy in killing cancer cells [1,2]. However, DOX can also cause serious and potentially irreversible heart damage, known as cardiotoxicity [3,4,5]. DOX-induced cardiotoxicity (DICT) can lead to life-threatening complications, including heart failure, arrhythmias, and other cardiac dysfunctions [6,7]. Despite extensive research, the precise mechanisms underlying DICT remain unclear. Current evidence suggests that oxidative stress, inflammation, and mitochondrial dysfunction play central roles in DOX-induced cardiac damage [8,9,10,11,12].

Furthermore, recent studies have shown that ferroptosis, a distinct form of regulated cell death, possibly contributes to DICT [13,14,15]. Ferroptosis, characterised by iron-dependent accumulation of lipid peroxides, leads to cellular membrane damage and death, particularly in highly oxidative cells, including cardiomyocytes [6,16,17,18,19,20]. Unlike apoptosis and necroptosis, ferroptosis does not involve the activation of caspase or the release of inflammatory molecules. Instead, it is initiated by intracellular oxidant depletion or inhibition of antioxidant enzymes, leading to reactive oxygen species (ROS) accumulation and subsequent lipid peroxidation [21,22,23,24].

Ferroptosis contributes to various cardiac conditions, including atherosclerosis, myocardial infarction, myocardial ischaemia–reperfusion injury, cardiomyopathy, and cardiac arrhythmias [25,26,27,28]. A previous study utilizing a murine DICT model demonstrated that ferrostatin-1 (Fer-1), a ferroptosis inhibitor, significantly reduced cardiac injury severity [13]. Additionally, other ferroptosis inhibitors, such as liproxstatin-1 (Lip-1), have shown protective effects against lipid peroxidation and mitochondrial dysfunction in cardiac cells [29,30]. These findings highlight ferroptosis as a key player in heart diseases and suggest that targeting ferroptosis may offer potential therapeutic strategies to alleviate disease progression. However, increasing evidence indicates that DICT cannot be effectively mitigated by blocking a single pathway, as multiple stress and cell-death pathways are simultaneously activated [31,32]. Therefore, there is a need to evaluate ferroptosis inhibitors across diverse experimental models to better define their therapeutic relevance in DICT.

In this study, we investigated the role of ferroptosis inhibitors in acute DICT in the heart by conducting multiple experimental models to evaluate their effects. First, we treated H9c2 cell lines with DOX and subsequently with ferroptosis inhibitors to assess their effects. Next, we used induced pluripotent stem cell (iPSC)-derived cardiomyocytes (iPSC-CMs) to examine the electrophysiological phenotypes associated with ferroptosis following DOX and ferroptosis inhibitors treatment. Finally, we employed a DOX-treated mouse model to investigate the molecular mechanisms of ferroptosis and evaluate the effects of ferroptosis inhibitors in vivo. Through these approaches, we aimed to elucidate the potential therapeutic effects of ferroptosis inhibitors in alleviating acute DICT.

## 2. Materials and Methods

### 2.1. Animals

Male C57BL/6J mice were obtained from Central Lab Animal, Inc. (Seoul, Korea) and maintained under a 12-h light/dark cycle with ad libitum access to food and water. This study complied with the Guide for the Care and Use of Laboratory Animals (National Institutes of Health Publication, 8th Edition, 2011) and the Institutional Animal Care and Use Committee of Yonsei University College of Medicine (Seoul, Korea) guidelines, with all animal experiments approved by the Institutional Animal Care and Use Committee of Yonsei University College of Medicine (Seoul, Korea) (permit number: 2019-0193). A total of 48 male C57BL/6J mice were used in this study (*n* = 8 per group for function and biochemical analysis; *n* = 4 per group for ECG recordings). All animals were of the same age, sex, and genetic background, and were housed under identical environmental conditions (temperature, light/dark cycle, and diet) to minimize potential confounding factors. The sample size for each group was determined based on prior studies of comparable design and outcome variability, ensuring adequate statistical power to detect a 25–30% difference in cardiac injury parameters between groups. The primary outcome measure used to determine sample size was cardiac injury, evaluated by echocardiographic and histological parameters.

### 2.2. In Vivo Mouse Cardiotoxicity Model

Twelve-week-old C57BL/6J wild-type littermate mice were used for all experiments. Mice were randomly assigned to four experimental groups; Vehicle, DOX only, DOX + Fer-1, and DOX + Lip-1. Randomization was performed manually at the time of group assignment. Ferrostatin-1 (Fer-1, 2 mg/kg) or Liproxstatin-1 (Lip-1, 10 mg/kg) was administered by intraperitoneal injection 24 h before DOX treatment (20 mg/kg, intraperitoneal). DOX was injected once on day 1. Echocardiography was performed on day 3, and ECG signals were continuously recorded throughout the study period, with representative data analyzed on days 1 and 21. Each animal represented a single experimental unit, as all measurements were obtained independently from individual mice. No animals were excluded from the analysis, and investigators were aware of group allocation during treatment, data collection, and outcome assessment.

### 2.3. Reagents

DOX was purchased from ChemScene (Princeton, NJ, USA). Fer-1 and Lip-1 were purchased from Selleck Chemicals (Houston, TX, USA). Detailed information on the antibodies used is provided in Supplementary Table S1.

### 2.4. H9c2 Cell Culture

The rat myoblast cell line (H9c2; RRID: CVCL_0286) was obtained from the American Type Culture Collection (Manassas, VA, USA). Cells were maintained in Dulbecco’s modified Eagle medium (Gibco-RBL, Waltham, MA, USA) supplemented with 10% fetal bovine serum (Gibco-RBL) and 1% antibiotic-antimycotic solution (Gibco-RBL). Cultures were incubated at 37 °C in a humidified atmosphere containing 5% CO_2_.

### 2.5. Human iPSCs Culture

Human induced pluripotent stem cells (hiPSCs) derived from normal human cord blood (CMC-hiPSC-011; RRID: CVCL_WR33) were provided by the National Stem Cell Bank of Korea (Korea National Institute of Health, Cheongju, Korea). Originally established by the Catholic University (Seoul, Korea), these hiPSCs were cultured in TeSR-E8 medium (Stemcell Technologies, Vancouver, BC, Canada) on plates coated with vitronectin. Cells were passaged every 4 days using ReLeSR reagent (Stemcell Technologies), following the manufacturer’s protocol.

### 2.6. Differentiation into Cardiomyocytes

Differentiation of iPSCs into cardiomyocytes was performed using a previously established protocol [33]. In brief, cells were seeded onto Matrigel-coated six-well plates and grown to approximately 90% confluence. To initiate mesoderm differentiation, cells were exposed to RPMI 1640 medium (Gibco-RBL) supplemented with B-27 minus insulin (Gibco-RBL) and 10 μM CHIR99021 (Tocris, Minneapolis, MN, USA) to activate Wnt/β-catenin signaling. After 48 h, the medium was replaced with RPMI 1640 containing B-27 minus insulin and a Wnt signaling inhibitor (C59; Selleck Chemicals) for another two days. The medium was then switched to RPMI 1640 supplemented with B-27 (Gibco-RBL) to promote cardiac maturation. For metabolic purification of cardiomyocytes, glucose deprivation was employed using RPMI 1640 without glucose supplemented with B-27 with insulin, as previously described [34].

### 2.7. Cell Viability Assay

H9c2 cells and iPSC-CMs were plated in 24-well plates (Corning Costar, Corning, NY, USA). Subsequently, EZ-Cytox solution (DoGENBio, Seoul, Korea) was added to each well, followed by a 2-h incubation. Cell viability was determined by measuring the absorbance at 450 nm. For the MTT assay, MTT solution (0.5 mg/mL, M2128, Sigma-Aldrich, St. Louis, MO, USA) was added to each well and incubated for 30 min at 37 °C. The resulting formazan crystals were dissolved in DMSO, and absorbance was measured at 570 nm. Relative viability was calculated by normalizing to an untreated control.

### 2.8. Immunohistochemistry

Mouse heart tissue was fixed in 4% buffered paraformaldehyde, embedded in paraffin, and cut into 4–5-µm-thick sections, which were stained with hematoxylin and eosin. Masson’s trichrome staining was performed using a Masson’s trichrome kit (StatLab, American MasterTech, Lodi, CA, USA) according to the manufacturer’s instructions. Briefly, embedded sections were de-paraffinized, rinsed with 100% ethanol and water, and incubated with Bouin’s fluid at 4 °C for 1 h. Subsequently, they were sequentially treated with Weiger’s working hematoxylin, Biebrich scarlet acid fuchsin, phosphomolybdic/phosphotungstic acid, aniline blue stain, and 1% acetic acid. Finally, the slides were dehydrated and mounted. Cardiac tissue fibrosis was assessed using a fluorescent microscope (IX71; Olympus, Tokyo, Japan).

### 2.9. Apoptosis Assay

H9c2 cells and iPSC-CMs were treated with DOX at 37 °C for 24 h. The cells were washed twice with phosphate-buffered saline (PBS) and resuspended in Annexin V-binding buffers (422201, BioLegend, San Diego, CA, USA). Subsequently, cells were harvested and stained with Annexin V-fluorescein isothiocyanate (640906, BioLegend) and Zombie NIR^TM^ fixable dye (423105, BioLegend) and incubated in the dark at room temperature for 15 min. Approximately 10^5^ cells per sample were analyzed using an LSR II flow cytometer (BD Bioscience, Franklin Lakes, NJ, USA) and the FlowJo v10.0.7 software (Tree Star Inc., Ashland, OR, USA).

### 2.10. Multi-Electrode Array Recording and Analysis

iPSC-CMs were seeded onto a 50 mg/mL fibronectin-coated CytoView multi-electrode array (MEA) 24-well plate (Axion Biosystems, Zurich, Switzerland) at a density of 50,000 cells per well, 7 days before the assays. Activity was recorded before treatment (baseline), immediately after DOX treatment (0 h), and 6 h post-treatment with DOX, Fer-1, and Lip-1 using the Maestro Edge MEA system (Axion Biosystems). Dimethyl sulfoxide was used as the vehicle control, and an equal volume of vehicle control or DOX was added to the wells. Signals were filtered using a bandpass filter ranging from 0.1 Hz to 2 kHz, and the beat detection threshold was set at 300 µV. Field potentials (FPs) were analyzed using the platform software, with output parameters including beat period (s), spike amplitude (µV), and FP duration (FPD, ms). Raw FPD measurements were corrected using Fredericia’s rate-correction algorithm (FPDcF), where FPDcF = FPD/Beat Period^0.33^. All recordings were captured using the standard cardiac settings (Axion Biosystems Maestro AxIS software version 2.1.1.5) at 37 °C.

### 2.11. NT-proBNP Measurement

Circulating NT-proBNP levels were measured using the NT-proBNP ELISA Kit (NBP2-76775, Novus Biologicals, Centennial, CO, USA) according to the manufacturer’s instructions. Briefly, plasma samples and standards were loaded into an antibody-coated 96-well microplate, followed by incubation with detection reagents and colorimetric development. Absorbance was measured at 450 nm using a microplate reader, and NT-proBNP concentrations were calculated from a standard curve.

### 2.12. Telemetry System

Twelve-week-old male mice were anaesthetised using isoflurane and implanted with a telemetry transmitter (EA-F20, Data Sciences International, St. Paul, MN, USA). Electrocardiogram (ECG) leads were arranged in a lead II configuration, with the negative lead positioned in the upper right chest and the positive lead placed below the left diaphragm and under the heart in the left abdomen. ECG recordings were conducted for 22 days, with each mouse housed individually in a cage equipped with a receiver (RPC-1, Data Sciences International). The analogue ECG signal was digitised using a 16-bit analogue-to-digital converter (HAI-118, iWorx, Dover, NH, USA) connected to a personal computer and captured using LabScribe software version 4.360 (iWorx).

### 2.13. Statistical Analysis

Statistical analyses were conducted using GraphPad Prism software version 8 (GraphPad Software, Boston, MA, USA). Data was presented as the mean ± standard error of the mean. For comparisons between two groups, an unpaired *t*-test was used for normally distributed data, while the Mann–Whitney U test was used for non-parametric data. When analyzing more than two groups, a one-way analysis of variance followed by Sidak’s multiple comparison test was applied for normally distributed data, while the Kruskal–Wallis test was used for non-parametric data. A *p*-value of <0.05 was considered statistically significant. For in vitro experiments, data represent biological replicates from at least three independent experiments (*n* = 3), and each experiment included three technical replicates per condition. For in vivo experiments, n represents the number of individual mice per group, as indicated in the figure legends.

## 3. Results

### 3.1. Effect of Ferroptosis Inhibitors on DOX-Induced Toxicity in H9c2 Cells

**Figure 1:**
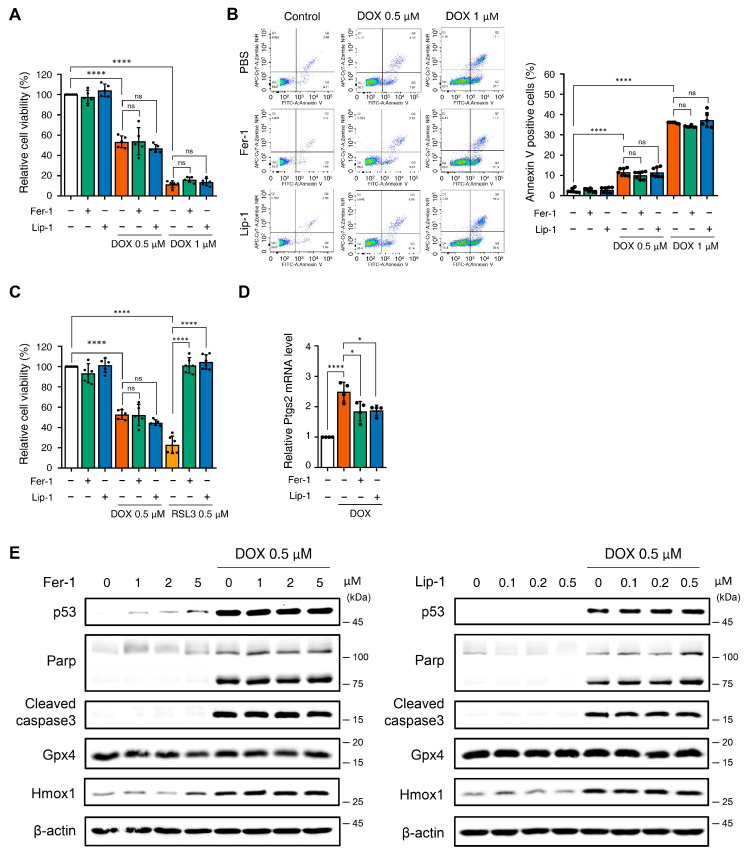
The application of ferroptosis inhibitors does not mitigate toxicity in H9c2 cells following treatment with doxorubicin. (**A**) Relative cell viability of H9c2 cells as determined by EZ-Cytox assay. H9c2 were pre-treated with ferrostatin-1 (Fer-1) and liproxstatin-1 (Lip-1) at concentrations of 2 and 0.2 μM, respectively, for 24 h. Additionally, doxorubicin was treated at concentrations of 0.5 and 1 μM for 24 h. **** *p* < 0.001. (**B**) Effect of Fer1, Lip-1, and/or doxorubicin treatment on apoptosis in H9c2 cells. Apoptosis was quantified by flow cytometry, measuring the percentage of annexin V-positive H9c2 cells. The fluorescence intensity of annexin V is plotted on the x-axis, and Zombie NIR is plotted on the y-axis. The percentage of apoptotic cells was investigated by using annexin V/Zombie NIR staining. Each bar is represented as the mean ± standard deviation. **** *p* < 0.001. (**C**) Relative cell viability of H9c2 cells following co-treatment with Fer-1, Lip-1, doxorubicin, and RSL3. H9c2 cells were pre-treated with Fer-1 and Lip-1 at concentrations of 2 and 0.2 μM, respectively, for 24 h. Additionally, doxorubicin and RSL3 were used at a concentration of 0.5 μM each for 24 h. (**A**–**C**), *n* = 5 independent experiments, **** *p* < 0.001. (**D**) Relative Ptgs2 mRNA levels treated with DOX in the presence or absence of ferroptosis inhibitors (Fer-1 or Lip-1) in H9c2 cells. Data are presented as mean ± S.D. (*n* = 4 independent experiments), normalized to Gapdh. * *p* < 0.05, **** *p* < 0.001. (**E**) Western blot analysis of apoptotic and ferroptotic cell death markers in H9c2 cells treated with doxorubicin. H9c2 cells were pre-treated with the indicated concentration of Fer-1 (left panel) or Lip-1 (right panel) for 24 h. One day after H9c2 cells were treated with 0.5 μM doxorubicin for 24 h. Data are presented as mean ± S.D. from biological replicates (*n* = 3 independent experiments). One-way ANOVA followed by Sidak’s multiple comparison test was used for (**A**–**C**), and the Kruskal–Wallis test was used for panel (**D**).

Recent studies suggest that ferroptosis inhibition mitigates H9c2 cytotoxicity induced by certain anticancer drugs, such as Herceptin, and protects against DICT [13,35]. We examined the effects of ferroptosis inhibitors (Fer-1 and Lip-1) in DOX-induced cell death in H9c2 cells to determine whether ferroptosis inhibition affects DICT. Surprisingly, DOX reduced cell viability in a dose-dependent manner; however, neither Fer-1 nor Lip-1, when co-administered individually with DOX, restored H9c2 cell viability (Figure 1A).

To verify these findings, we tested higher concentrations of Fer-1 and Lip-1 after DOX treatment. However, no improvement in cell viability was observed at any tested dose (Supplementary Figure S1A,B). Apoptosis analysis using Annexin V/Zombie NIR staining and flow cytometry consistently confirmed that Fer-1 and Lip-1 did not reduce the apoptotic cell proportions (Figure 1B).

Fer-1 and Lip-1 effectively prevented RSL3-induced cell death; however, they failed to protect against DOX-induced cytotoxicity in H9c2 cells (Figure 1C and Supplementary Figure S1C). To further evaluate ferroptosis-associated responses, we quantified Ptgs2 mRNA expression, which is among the most commonly assessed transcriptional markers in ferroptosis research. DOX treatment increased Ptgs2 expression in H9c2 cells, indicating activation of ferroptosis-associated transcriptional signaling (Figure 1D). In addition, Western blot analysis showed that 4-HNE levels were elevated after DOX exposure and were reduced by Fer-1 and Lip-1 (Supplementary Figure S1D).

Western blot analysis was conducted to investigate the effects of Fer-1 and Lip-1 on DOX- and RSL3-induced cell death pathways. DOX-treated H9c2 cells exhibited increased expression of markers associated with p53-dependent apoptosis (p53, cleaved poly (adenosine diphosphate-ribose) polymerase, and cleaved caspase-3), as well as the ferroptosis-related marker Heme Oxygenase 1 (Hmox1). Notably, Fer-1 and Lip-1 did not attenuate these changes in DOX-treated cells. Conversely, the RSL3-induced glutathione peroxidase 4 (GPX4) depletion was partially reversed by Fer-1 treatment, confirming its efficacy in targeting ferroptosis (Figure 1E and Supplementary Figure S2).

### 3.2. Ferroptosis Inhibitors on DOX-Induced Toxicity or Electrophysiological Dysfunction in iPSC-CMs

**Figure 2:**
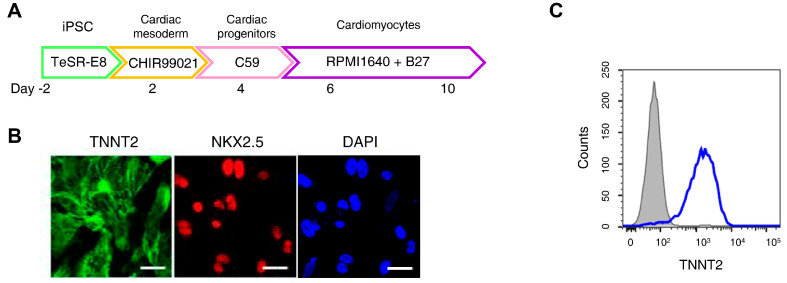

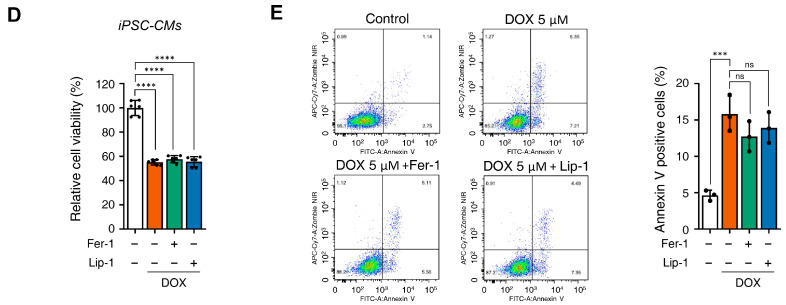
Ferroptosis inhibitors do not alleviate doxorubicin-induced toxicity in induced pluripotent stem cell (iPSC)-derived cardiomyocytes (CMs). (**A**) Schematic representation of the iPSC-CMs differentiation. The validated hiPSCs were cultured in the indicated media during the differentiation period and finally differentiated to mature hiPSC-CMs through cardiac mesoderm, cardiac progenitors, and cardiomyocytes. (**B**) To validate iPSC-CMs, cells were stained with the indicated antibodies recognizing cardiac markers (Troponin T2, NK2 Homeobox 5), and immunofluorescence was analyzed; Scale bar = 100 µm. (**C**) Troponin T2-positive cells were assessed using flow cytometry. (**D**) Relative cell viability of iPSC-CMs as determined by EZ-Cytox assay. iPSC-CMs were pre-treated with ferrostatin-1 (Fer-1) and liproxstatin-1 (Lip-1) at concentrations of 2 and 0.2 μM, respectively, for 24 h. Additionally, doxorubicin was treated at concentrations of 5 μM for 24 h. (**E**) Flow cytometric quantification of apoptosis in iPSC-CMs treated with Fer-1, Lip-1, and/or doxorubicin. Fer-1 or Lip-1 was applied to iPSC-CMs, and the cells were exposed to 5 µM doxorubicin for 24 h Apoptosis was quantified by flow cytometry, measuring the percentage of Annexin V-positive cells. The percentage of apoptotic cells was detected using annexin V/Zombie NIR staining and analyzed using flow cytometry. A representative image of fouFr independent experiments is shown (left panel). The histogram depicts the quantification of apoptosis (in percentage) for treatments (right panel). Data are presented as mean ± S.D. from biological replicates (*n* = 3 independent experiments). ns, no significance. *** *p* < 0.005, **** *p* < 0.001. One-way ANOVA followed by Sidak’s multiple comparison test was used for panel (**D**), and the Kruskal–Wallis test was used for panel (**E**).

H9c2 cells are widely used in cardiomyocyte research; however, they do not fully reproduce human cardiomyocyte characteristics. An increasing number of studies have employed iPSC-CMs to better model human cardiac physiology [36]. Figure 2A presents an overview of the iPSC-CMs differentiation process. Immunofluorescence staining confirmed high expression of cardiomyocyte markers Troponin T2 and NK2 Homeobox 5 in differentiated iPSC-CMs, while flow cytometry analysis showed that over 90% of the cells expressed Troponin T2 (Figure 2B,C).

Cell viability assays demonstrated that, similarly to H9c2 cells, iPSC-CMs did not recover from DOX-induced cell death upon treatment with Fer-1 or Lip-1 (Figure 2D). Additionally, flow cytometry showed that DOX-induced cell death of iPSC-CMs remained unchanged upon Fer-1 or Lip-1 treatment (Figure 2E). These findings suggest that, as observed in H9c2 cells, ferroptosis inhibitors do not protect iPSC-CMs against DOX-induced cytotoxicity.

**Figure 3:**
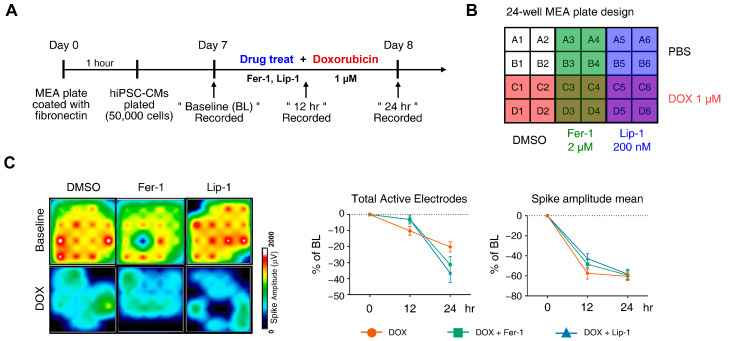

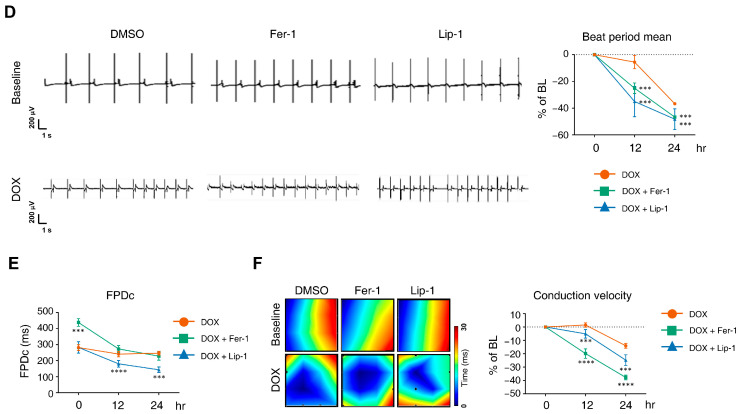
Electrophysiological alterations of induced pluripotent stem cell (iPSC)-derived cardiomyocytes (CMs) induced by doxorubicin treatment are not ameliorated by ferroptosis inhibitors. (**A**) Schematic diagram of drug and doxorubicin treatment experiments. (**B**) Plate schematic for seeding iPSC-CMs to perform a multi-electrode assay. (**C**) An activity map of the total active electrode count is shown. The red and blue colors indicate higher and lower spike amplitudes for iPSC-CMs, respectively. Representative images are shown from three independent experiments (left panel). The percentage of total active electrodes and the percentage changes in spike amplitude compared to the baseline were measured using a multi-electrode array (MEA) assay (right two panels). (**D**) Field potential trace data recorded with the MEA, exhibiting mean spontaneous beating traces, are shown. Representative trace images recorded with the MEA display field potential traces in iPSC-CMs treated with ferroptosis inhibitors with or without DOX (left six panels). The graph indicates beat period and spike amplitude measured across the treatment groups (right panel). (**E**) The graph displays field potential duration corrected (FPDc) data obtained from field potential traces recorded with the MEA, illustrating the effects of ferroptosis inhibitors with or without DOX on iPSC-CMs. (**F**) Conduction plot showing the propagation delay of iPSC-CMs with ferroptosis inhibitors and doxorubicin. The blue region represents the origin of the beat (start electrode). Different colors represent the propagation delay time, as shown in the scale bar. Representative images are demonstrated from three independent experiments (left panel). The percentage change compared to the baseline was measured using MEA (right panel). Data are presented as mean ± S.D. from biological replicates (*n* = 3 independent experiments), each recorded from multiple technical electrodes per condition. Statistical significance was determined by one-way ANOVA followed by Sidak’s multiple comparison test (*** *p* < 0.005, and **** *p* < 0.001).

DOX has been linked to various arrhythmias in humans, including sinus tachycardia, atrial fibrillation, T-wave flattening, and QT interval irregularities [37,38,39,40]. We performed MEA analyses on hiPSC-CMs at 12 and 24 h post-treatment with DOX and a ferroptosis inhibitor. MEA recordings were conducted 7 and 8 days after seeding differentiated iPSC-CMs onto an MEA plate to establish a baseline measurement before drug treatment to assess subsequent electrophysiological responses (Figure 3A). Each experimental group was performed in four wells following the plate map design (Figure 3B).

DOX treatment led to a reduction in active electrode numbers (>200 μV for spike threshold) per well and spike amplitude at both 12 and 24 h, reducing the number of detected electrodes (Figure 3C). However, this effect was not rescued with ferroptosis inhibitors. Additionally, DOX induced a time-dependent reduction in the beat period of iPSC-CMs, which was further accelerated by ferroptosis inhibitor treatment (Figure 3D). Ferroptosis inhibitors also increased beat period irregularity in DOX-treated iPSC-CMs. The FPD–corrected (FPDc), a parameter reflecting action potential repolarization, was assessed (Figure 3E,F). Both FPDc and conduction velocity decreased in the ferroptosis inhibitor-treated cells, indicating an increased electrophysiological vulnerability. FPDc changes were independent of ferroptosis inhibitor treatment; however, DOX-induced conduction velocity reduction was further exacerbated by ferroptosis inhibitors in a time-dependent manner (Figure 3F).

### 3.3. Ferroptosis Inhibitors on Cardiac Dysfunction Rescue

**Figure 4:**
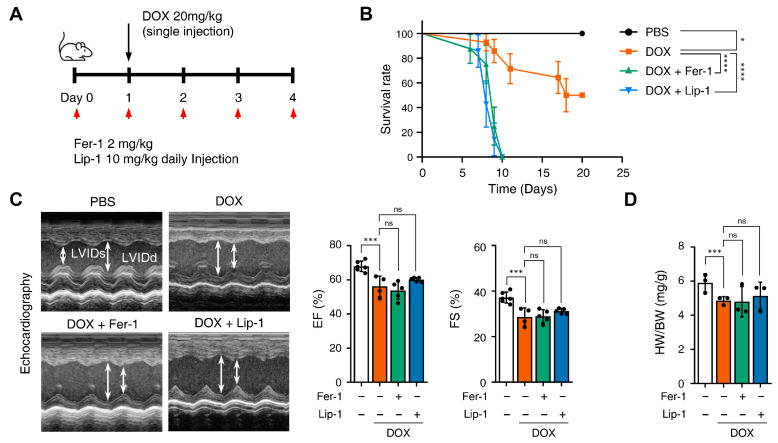

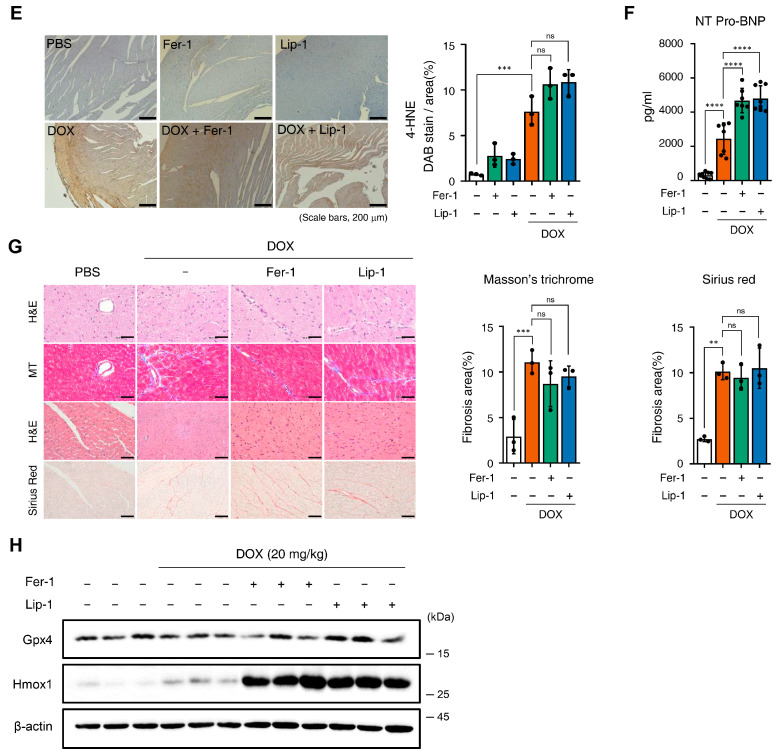
Ferroptosis inhibitors fail to rescue cardiac dysfunction. (**A**) Brief schedule of in vivo experiment. C57BL/6J mice were treated with ferrostatin-1 (Fer-1) or liproxstatin-1 (Lip-1) 24 h before doxorubicin injection and were dosed every 24 h. A single dose of doxorubicin was administered intraperitoneally once on day 1. Experimental groups: Vehicle, DOX (20 mg/kg, i.p.), DOX + Fer-1 (2 mg/kg, i.p.), and DOX + Lip-1 (10 mg/kg, i.p.) (**B**) The survival graph of mice from different groups was shown by Kaplan–Meier survival analysis. (**C**) Three days after intraperitoneal injection of doxorubicin, the mouse cardiac function was analyzed with echocardiography. The echocardiography data showed decreased fractional shortening and ejection fraction in mice treated with doxorubicin and Fer-1 or Lip-1. Survival rate, *n* = 5 per group; ECG, *n* = 5 per group. (**D**) Bar graph representation of the heart weight to body weight ratio of mice. (**E**) 3,3’-Diaminobenzidine immunohistochemical staining for 4-hydroxynonenal (4-HNE) in the mouse heart tissue; scale bars, 200 µm (left panel). Quantification of 4-HNE-specific 3,3’-Diaminobenzidine staining (right panel). (**F**) Bar graph representation of the N-terminal pro B-type natriuretic peptide (NT-proBNP) of mice. (**G**) Masson’s trichrome and Sirius red staining of fibrosis in the mouse heart (left panel) and quantification (right panel). (**H**) Western blot of ferroptosis cell death markers in mouse heart tissues treated with doxorubicin and Fer-1 or Lip-1. heart weight to body weight, *n* = 3 per group; 4-HNE, *n* = 3 per group; NT-proBNP, *n* = 7 per group; Masson’s trichrome and Sirius red, *n* = 3 per group; Western blots, *n* = 3 per group. Data are presented as mean ± S.D., where n represents the number of individual mice per group. Statistical significance was determined by one-way ANOVA followed by Sidak’s multiple comparison test for panels (**C**,**F**), and the Kruskal–Wallis test was used for panels (**D**,**E**,**G**), ns, no significance, * *p* < 0.05, ** *p* < 0.01, *** *p* < 0.005, **** *p* < 0.001.

Fer-1 has been suggested as a potential cardioprotective agent against DICT. Therefore, we investigated whether Fer-1 and Lip-1 exert cardioprotective effects using a mouse model of DICT. Figure 4A provides a schematic illustration of the injection schedule: Fer-1 (2 mg/kg) and Lip-1 (10 mg/kg) were administered intraperitoneally once daily, while DOX (20 mg/kg) was injected 1 day after the first Fer-1/Lip-1 injection.

We compared mice survival in the PBS, DOX + Fer-1, and DOX + Lip-1 experiment groups. Unexpectedly, the survival rate was significantly lower in the groups treated with both DOX and Fer-1 or Lip-1 than in the DOX-only group (Figure 4B). To investigate whether the accelerated mortality was due to damage in organs other than the heart, comprehensive histological and biochemical evaluations were conducted on the kidney, liver, lung, and spleen. The results revealed no significant abnormalities in these organs (Supplementary Figure S3). Although a reduction in the white pulp area of the spleen was observed following ferroptosis inhibitor treatment (Supplementary Figure S3A), the extent of this change was insufficient to impact survival rates. Moreover, biochemical analyses demonstrated elevated alanine aminotransferase (ALT) levels (Supplementary Figure S3C), which is consistent with DOX-induced hepatotoxicity reported in previous studies [41,42].

Echocardiography was used to further assess the effects of these treatments, and blood samples were collected 4 days after DOX injection, as illustrated in Figure 4A. Echocardiography revealed impaired cardiac function in DOX-treated mice compared with PBS-treated mice. Both ejection fraction and fractional shortening were significantly reduced in DOX-treated mice compared to PBS-treated mice and were not restored with Fer-1 or Lip-1 pretreatment (Figure 4C). Additionally, the heart-weight-to-body ratio was lower in DOX-treated mice than in PBS-treated mice and, similar to the echocardiography results, was not restored with Fer-1 or Lip-1 pretreatment (Figure 4D).

Furthermore, we performed immunostaining for 4-hydroxynonenal (4-HNE), a well-established marker of ferroptosis-associated lipid peroxidation, to evaluate cardiac tissue ferroptosis. DOX treatment increased 4-HNE expression, which was not reduced with Fer-1 or Lip-1 treatment. Interestingly, a slight increase in 4-HNE expression was also observed in the hearts of mice treated with Fer-1 or Lip-1 alone compared with controls (Figure 4E).

Pro B-type Natriuretic Peptide (proBNP) is a protein predominantly produced by the left ventricular cells of the heart and is present in the blood as the active hormone brain natriuretic peptide (BNP) and the inactive N-Terminal (NT)-proBNP fragment. NT-proBNP is a sensitive marker of left ventricular function and is upregulated in heart failure, making it a diagnostic and prognostic marker for heart failure [43]. Interestingly, DOX injection significantly increased NT-proBNP levels in the blood, and this effect was further amplified with Fer-1 or Lip-1 treatment (Figure 4F).

Masson’s trichrome and Sirius Red staining were performed to assess the extent of cardiac fibrosis. Fer-1 or Lip-1 pretreatment did not reduce DOX-induced cardiac fibrosis (Figure 4G), indicating that these treatments lacked cardioprotective effects. Additionally, proteins were purified from the hearts of three mice in each group, and Gpx4 and Hmox1 (representative genes of ferroptosis-induced expression changes) expression levels were assessed. Gpx4 expression remained unchanged in all groups, while Hmox1 expression was increased with DOX injection and was further amplified with Fer-1 or Lip-1 addition (Figure 4H).

To further investigate whether ferroptosis inhibition alone is insufficient to prevent DOX-induced cardiotoxicity, we performed additional cell viability assays in H9c2 cells using inhibitors targeting multiple regulated cell-death pathways. Interestingly, results showed that neither Fer-1, the necroptosis inhibitor GSK’963, nor the pan-caspase inhibitor Z-VAD-FMK alone restored cell viability under DOX treatment. In contrast, co-inhibition of ferroptosis with apoptosis or necroptosis alleviated DOX-induced cytotoxicity (Supplementary Figure S4).

Taken together, these results demonstrate that ferroptosis inhibition alone under acute DOX exposure conditions does not prevent DICT. Instead, DICT likely involves multiple regulated cell-death pathways simultaneously.

### 3.4. Ferroptosis Inhibitors on DOX-Induced Arrhythmic Changes in Mice

**Figure 5:**
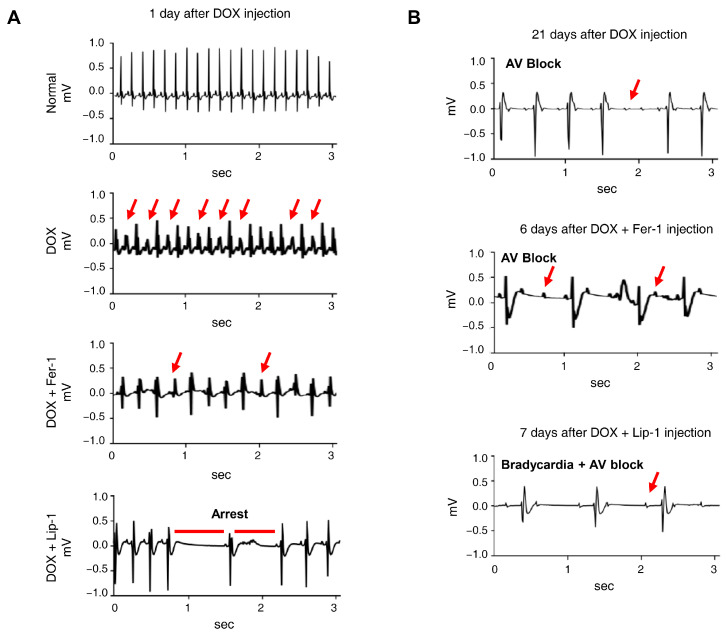
Ferroptosis inhibitors do not alleviate doxorubicin-induced arrhythmic changes in mice. (**A**) Electrocardiogram (ECG) traces recorded on day 1 after DOX administration with or without ferroptosis inhibitors, showing various arrhythmic events, including tachyarrhythmia, frequent premature ventricular contractions, irregular rhythm, and cardiac arrest. Red arrows indicate representative arrhythmic events, including premature ventricular contractions and irregular R−R intervals. (**B**) ECG traces recorded on day 21, demonstrating the progression of arrhythmic changes, including atrioventricular (AV) block and bradycardia with AV block, indicated by red arrows. *n* = 4 per group. Data are presented as mean ± S.D., where n represents the number of individual mice per group. Statistical significance was determined by one-way ANOVA followed by Sidak’s multiple comparison test.

Electrocardiograms (ECGs) are particularly valuable for detecting early onset of cardiotoxic effects and preclinical cardiac damage [44]. Our electrophysiological analysis of iPSC-CMs revealed that the co-administration of DOX and a ferroptosis inhibitor worsened electrophysiological parameters. The ECG was continuously monitored for 24 h in conscious, freely moving mice using implanted telemetry devices to assess the effects of DOX and ferroptosis inhibitors on cardiac function and mortality in vivo. Representative ECG images captured baseline and 7 days post-administration revealed the following: In the DOX-only group, ECG abnormalities were observed, with atrioventricular block appearing 21 days post-administration. In the cardiotoxicity model, abnormalities were noted at the start of Fer-1 administration, with atrioventricular block manifesting after 6 days. In the Lip-1-treated group, early administration led to cardiac arrest, followed by bradycardia and atrioventricular block after 7 days (Figure 5A,B).

## 4. Discussion

In this study, we investigated the effects of a ferroptosis inhibitor on acute DICT using cell lines, iPSC-CMs, and mouse models. Our findings demonstrated that while Fer-1 and Lip-1 effectively inhibited RSL3-mediated cell death, they did not prevent DOX-induced cytotoxicity in H9c2 cells. Using iPSC-CMs, we found that co-treatment with DOX and ferroptosis inhibitors further decreased electrical activity and exacerbated electrophysiological abnormalities. Finally, in the mouse acute DICT model, ferroptosis inhibitor administration significantly reduced survival rates and worsened cardiac outcomes, whereas other organs remained unaffected. These findings indicate that rather than providing cardioprotection, ferroptosis inhibitors may exacerbate DICT by worsening cardiac function and reducing survival rates.

DICT is widely recognised to involve mechanisms, including oxidative stress, inflammation, and direct toxicity to cardiomyocytes [45]. Among these, oxidative stress is the most widely accepted explanation, as DOX generates excessive ROS, which damages essential cellular components in cardiomyocytes [46]. Mitochondrial dysfunction is another key mechanism wherein DOX directly impairs mitochondrial electron transport chain activity, leading to increased ROS production and subsequent adenosine triphosphate depletion [47,48]. Additionally, DOX induces cardiomyocyte apoptosis through activation of the p53 pathway and cytochrome c release, which subsequently triggers caspase activation [49]. Pro-inflammatory responses involving the release of cytokines such as tumor necrosis factor-α and interleukin-6 are also implicated in DOX-induced cardiac injury, further exacerbating myocardial damage [50,51]. In addition to apoptosis, other forms of regulated cell death, including necroptosis, pyroptosis, and ferroptosis, have also been implicated in DICT [52,53,54,55,56,57]. These pathways often interact or coexist, contributing to the complex and multifactorial nature of DOX-induced cardiomyocyte injury. Emerging evidence has highlighted ferroptosis, distinct from apoptosis and necrosis, as a potential contributor to the complex interplay of cell death mechanisms involved in DICT [13]. Recent studies have shown that ferroptosis may amplify oxidative stress and contribute to DICT injury progression [58].

While previous studies suggest that ferroptosis is involved in DICT and that its inhibitors may have cardioprotective effects [13], our results showed a contrasting perspective. Specifically, we observed that Fer-1 and Lip-1 did not reduce DOX-induced toxicity in H9c2 cells, decrease cell death, or enhance electrophysiological stability in iPSC-CMs, nor did they prevent cardiac dysfunction or reduce arrhythmic changes in the mouse model. Therefore, our results suggest that ferroptosis inhibition alone may not be sufficient to mitigate DICT. This raises the possibility that DOX-induced cardiac damage may involve multiple overlapping cell death pathways, including apoptosis and necrosis, rather than relying solely on ferroptosis. These observations challenge previously reported cardioprotective effects of ferroptosis inhibitors in DOX-induced cardiac injury.

To explore alternative mechanisms, we considered apoptosis and necroptosis as potential contributors to DOX-induced cardiac injury. These programmed cell death pathways have been extensively documented in DICT and are closely linked to the interplay of oxidative stress, mitochondrial dysfunction, and inflammation [46,55]. Specifically, DOX is also known to induce apoptosis via mitochondrial pathways, such as p53 and Bax activation, or through necrosis triggered by excessive ROS production [59]. These observations collectively support the notion that DOX-induced cardiomyocyte death is governed by a network of cell death pathways rather than a single dominant mechanism. Accordingly, we further investigated the interplay between ferroptosis, apoptosis, and necroptosis by co-administering ferroptosis inhibitors with either the pan-caspase inhibitor Z-VAD-FMK or the necroptosis inhibitor GSK’963 in H9c2 cells. Notably, none of the inhibitors alone restored cell viability, whereas only the combined treatment of ferroptosis inhibition with apoptosis or necroptosis inhibition significantly rescued DOX-induced cell death (Supplementary Figure S4). This finding suggests that simultaneously modulating multiple RCD pathways may be more effective in mitigating DOX-induced toxicity than targeting a single pathway. Importantly, previous studies have shown that selective inhibition of apoptosis, such as with caspase-3 inhibitors, provides limited protection against DICT [31,60], reinforcing the idea that single-pathway interventions are insufficient.

Remarkably, in our mouse model, the group co-administered Fer-1 and Lip-1 with DOX exhibited a faster mortality rate than the group treated with DOX alone. This finding suggests that ferroptosis inhibitors not only fail to confer protective effects against the extensive side effects of DOX but may also interfere with other cellular pathways, potentially exacerbating its adverse effects on the heart. In both our in vitro and in vivo experiments, GPX4 protein levels remained largely unchanged following DOX exposure and were not restored by ferroptosis inhibitors. Although our findings differ from a previous report [13], several studies have similarly documented stable GPX4 expression under acute DOX-induced stress [61], indicating that GPX4 abundance does not necessarily reflect functional ferroptotic activity. Despite the lack of GPX4 modulation, 4-HNE—an indicator of lipid peroxidation—remained persistently elevated, and Western blot analysis revealed that Hmox1 expression was not only induced by DOX but further augmented upon ferroptosis inhibitor administration. However, 4-HNE is not a ferroptosis-specific marker but rather a downstream product of lipid peroxidation that can be generated through multiple cellular stress responses, including broadly under inflammatory and oxidative stress conditions, and is not restricted to ferroptosis-related lipid peroxidation [62,63]. Hmox1 expression is strongly induced by various chemical and physical stresses, including oxidative stress, heat shock, heme and hemin, inflammatory cytokines, lipopolysaccharides, hypoxia, and hydrogen peroxide [64,65,66,67,68]. One plausible explanation is that inhibition of lipid peroxidation diverts intracellular iron away from lipid peroxidation, thereby increasing the pool of free iron that fuels the Fenton reaction and exacerbates oxidative injury [69]. In this context, excessive ROS production, combined with limited antioxidant defences such as superoxide dismutase (SOD), catalase, and glutathione peroxidase, could intensify redox imbalance and ultimately amplify oxidative damage even in the presence of ferroptosis inhibitors [46,70]. Therefore, the paradoxical increase in oxidative stress observed with Fer-1 and Lip-1 treatment suggests that inhibition of ferroptosis alone is insufficient to prevent DICT in our model. Consistent with our findings, a recent study has shown that ferroptosis inhibition does not prevent myocardial injury in acute settings and may shift the injury response toward an inflammatory-dominant phenotype rather than providing cardioprotection [71]. This cardiac context likely reflects strong iron-dependent activation of multiple regulated cell-death pathways in cardiomyocytes.

This study has several limitations. First, we employed an acute DICT model, which may not fully capture the chronic and multifactorial mechanisms underlying DOX-induced cardiac injury. Second, although Fer-1 and Lip-1 reduced 4-HNE levels in vitro, we did not directly quantify their myocardial delivery in vivo. Third, our analyses focused primarily on the effects of ferroptosis inhibitors; consequently, potential actions of Fer-1 and Lip-1 on other regulated cell death mechanisms remain unexplored. Fourth, we did not evaluate the expression or enzymatic activity of antioxidant enzymes—including SOD and catalase—which could influence redox imbalance and Hmox1 induction in DICT. To address these limitations, future studies should systematically evaluate both acute and chronic DICT models across a range of DOX concentrations, together with dose–response analyses of Fer-1 and Lip-1, to better define the conditions under which ferroptosis inhibition may be protective. Direct confirmation of inhibitor delivery to cardiac tissue—such as through mass spectrometry–based quantification—will also be necessary to establish effective target engagement in vivo. In addition, comparative analyses of multiple programmed cell death pathways will be essential to delineate how ferroptosis interacts with other cell death mechanisms in the context of DOX-induced cardiotoxicity.

In conclusion, our findings demonstrate that Fer-1 and Lip-1 did not provide cardioprotective effects in acute DICT and may even exacerbate cardiac injury. These results suggest that ferroptosis inhibitors should be used with caution in the context of DICT. Notably, simultaneous inhibition of ferroptosis together with apoptosis or necroptosis provided greater protection against DICT than targeting any single pathway alone, underscoring the therapeutic potential of combinatorial approaches.

## Data Availability

The original contributions presented in this study are included in the article/Supplementary Materials. Further inquiries can be directed to the corresponding authors.

## Supplementary Materials

The following supporting information can be downloaded at: https://www.mdpi.com/article/10.3390/antiox15010027/s1, Figure S1: Cytotoxicity assessment and efficacy validation of ferroptosis inhibitors in H9c2 cells; Figure S2: Effects of ferroptosis inhibitor on ferroptosis and cell death pathways in H9c2 cells; Figure S3: Evaluation of organ toxicity in mice treated with doxorubicin and ferroptosis inhibitors; Figure S4: Co-inhibition of ferroptosis and apoptosis or necroptosis alleviates doxorubicin-induced cytotoxicity in H9c2 cells. Table S1: List of antibodies used in this study.

## Abbreviations

The following abbreviations are used in this manuscript:

Fer-1	Ferrostatin-1
Lip-1	Liproxstatin-1
DOX	Doxorubicin
DICT	DOX-induced cardiotoxicity
NT-ProBNP	N-terminal pro B-type natriuretic peptide
ROS	Reactive oxygen species
iPSC-CMs	Induced pluripotent stem cell-derived cardiomyocytes
Hmox1	Heme Oxygenase 1
Gpx4	Glutathione peroxidase 4
