# Supplementary material for: Limitations of Ferroptosis Inhibitors on the Doxorubicin-Induced Cardiotoxicity
Source: Antioxidants (Basel). 2025 Dec 24;15(1):27. doi: 10.3390/antiox15010027 (PMC12837370; doi:10.3390/antiox15010027)
Supplement: Supplementary file 1 [file antioxidants-15-00027-s001.zip › antioxidants-4018472-supplementary.pdf]

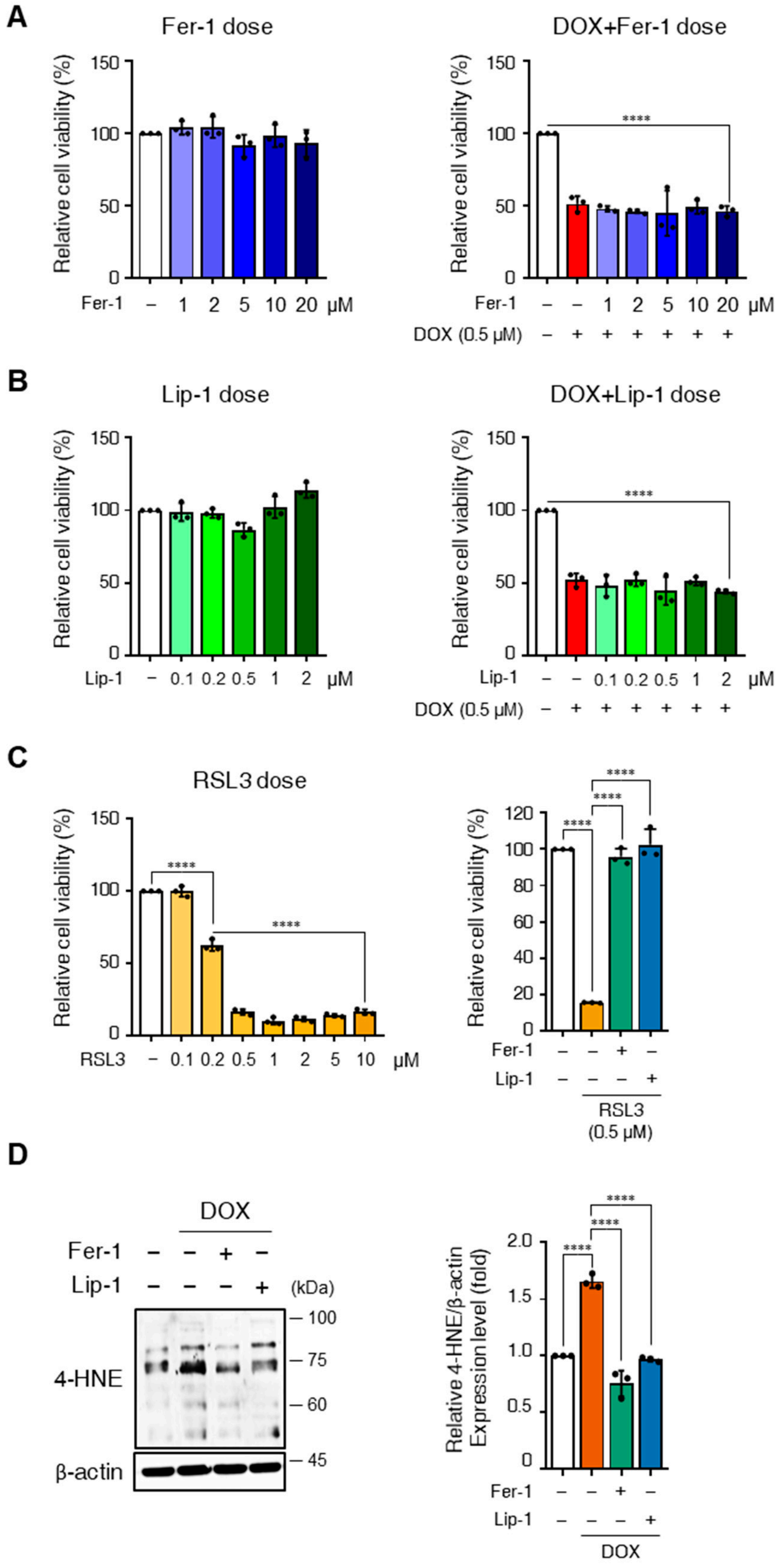

Supplementary figure 1. Cytotoxicity assessment and efficacy validation of ferroptosis inhibitors in H9c2 cells

A. MTT assay of cell viability in H9c2 cells treated with increasing doses of Ferrostatin-1 (Fer-1) (left panel) and co-treatment with Doxorubicin (0.5 μM) and Fer-1 (right panel); n=3 per group. **B.** MTT assay of cell viability in H9c2 cells treated with increasing doses of Ferrostatin-1 (Fer-1) (left panel) and co-treatment with Doxorubicin (0.5 μM) and Fer-1 (right panel); n=3 per group. **C.** MTT assay of cell viability in H9c2 cells treated with increasing doses of RSL3 (left panel) and co-treatment with RSL3 and ferroptosis inhibitors (Fer-1 or Lip-1) (right panel); n=3 per group. **D**. Western blot analysis of 4-HNE level in H9c2 cells treated with DOX (0.5 μM, 24 h) with or without ferroptosis inhibitors (Fer-1, 2 μM; Lip-1, 0.2 μM) (left panel), and quantification of the 4-HNE expression level normalized to β-actin (right panel). Statistical significance was determined by one-way ANOVA followed by Sidak's multiple comparison test. *$p < 0.05$; **$p < 0.01$; ***$p < 0.005$; ****$p < 0.001$.

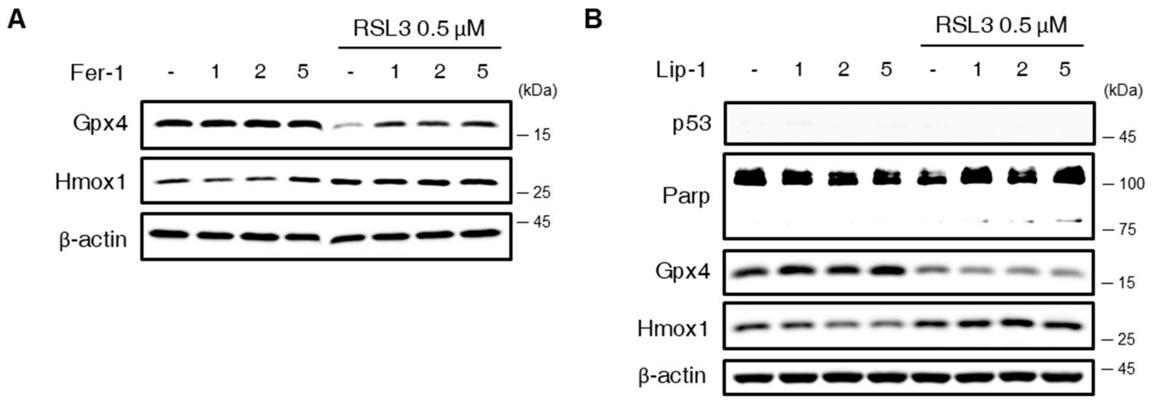

Supplementary figure 2. Effects of ferroptosis inhibitor on ferroptosis and cell death pathways in H9c2 cells
A. Western blot analysis of ferroptosis markers in H9c2 cells treated with Ferrostatin-1 (Fer-1) alone and co-treated with RSL3 and Fer-1. **B.** Western blot analysis of cell death markers and ferroptosis markers in H9c2 cells treated with Liproxstatin-1 (Lip-1) alone and in combination with RSL3.

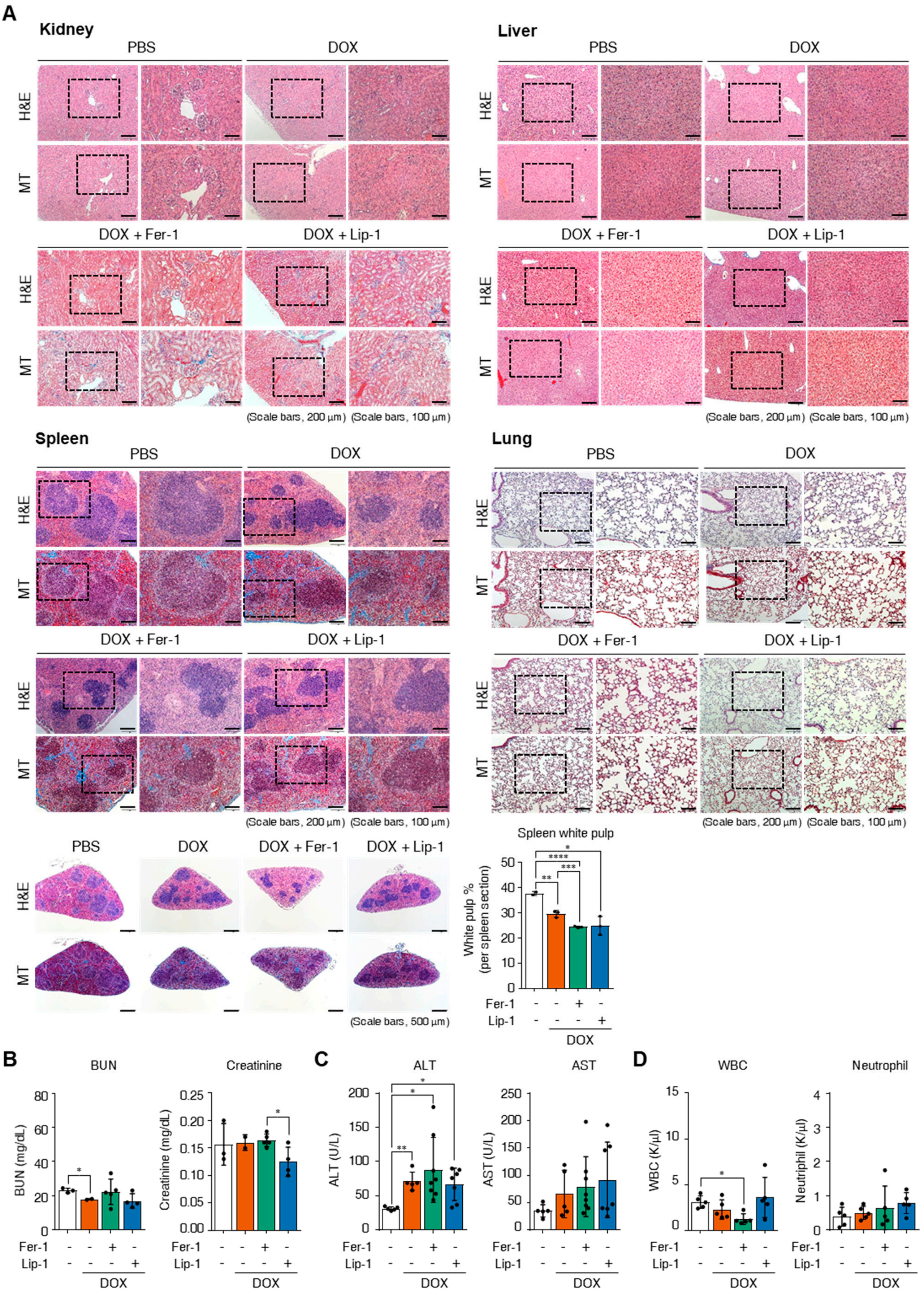
A
Kidney
Liver
PBS
DOX
DOX + Fer-1
DOX + Lip-1
H&E
MT
(Scale bars, 200 μm) (Scale bars, 100 μm)
Spleen
Lung
(Scale bars, 500 μm)
Spleen white pulp
White pulp % (per spleen section)
Fer-1
Lip-1
DOX
B
BUN
BUN (mg/dL)
Creatinine
Creatinine (mg/dL)
C
ALT
ALT (U/L)
AST
AST (U/L)
D
WBC
WBC (K/μl)
Neutrophil
Neutriphil (K/μl)

Supplementary figure 3. Evaluation of organ toxicity in mice treated with doxorubicin and ferroptosis inhibitors

A. Representative histological images of kidney, liver, spleen, and lung tissues from mice treated with phosphate-buffered saline (PBS, control), doxorubicin (DOX), DOX with ferrostatin-1 (DOX + Fer-1), or DOX with liproxstatin-1 (DOX + Lip-1). Tissues were stained with Hematoxylin and Eosin (H&E) and Masson's Trichrome (MT). Quantification of spleen white pulp area is shown; n=3 per group. **B.** Blood urea nitrogen (BUN) and creatinine levels in mice treated with PBS, DOX, DOX + Fer-1, or DOX + Lip-1. **C.** Alanine aminotransferase (ALT) and aspartate aminotransferase (AST) levels in mice treated with PBS, DOX, DOX + Fer-1, or DOX + Lip-1. **D.** White blood cell (WBC) count and neutrophil percentage in mice treated with PBS, DOX, DOX + Fer-1, or DOX + Lip-1. Statistical significance was determined by one-way ANOVA followed by Sidak's multiple comparison test. $^{*}p < 0.05$; $^{**}p < 0.01$; $^{***}p < 0.005$; $^{****}p < 0.001$.

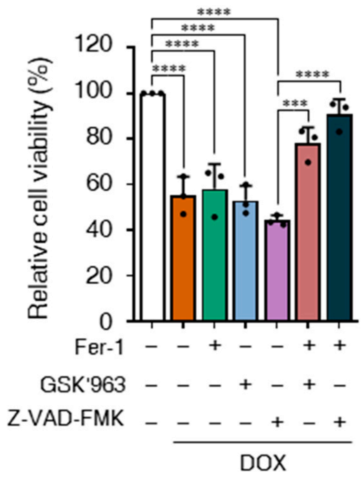

Supplementary figure 4. Co-inhibition of ferroptosis and apoptosis or necroptosis alleviates doxorubicin-induced cytotoxicity in H9c2 cells.

Cell viability of H9c2 cells treated with doxorubicin (0.5 μM) for 24 h in the presence of Ferrostatin-1 (Fer-1, 2 μM), necroptosis inhibitor (GSK'963, 5 μM), or pan caspase inhibitor (Z-VAD-FMK, 50 μM), either alone or in combination. H9c2 cells were pre-treated with Fer-1, GSK'963, or Z-VAD-FMK for 1 h prior to doxorubicin exposure. Data are presented as mean ± S.D. (n=3 independent experiments), and the Kruskal-Wallis test was used to compare groups.

Supplementary Table

Table 1. List of antibody used in this study

Target antigen	Vendor or Source	Catalog #	Dilution	Persistent ID / URL
p53	Cell Signaling Technology	2527	WB 1:1000	RRID : AB_10695803
Parp	Cell Signaling Technology	9542	WB 1:1000	RRID : AB_2160739
Clv-Caspase-3	Cell Signaling Technology	9661	WB 1:1000	RRID : AB_2341188
Gpx4	Cell Signaling Technology	52455	WB 1:1000	RRID : AB_2924984
Hmox1	Thermo Fisher	GT1334	WB 1:1000	RRID : AB_2787185
β-actin	Santa Cruz Biotechnology	sc-47778	WB 1:1000	RRID : AB_2714189
Alexa Fluor 488 Chicken anti-rabbit	Thermofisher	A21200	IF 1:500	RRID : AB_2535786
Alexa Fluor 546 goat anti-mouse	Thermofisher	A11030	IF 1:500	RRID : AB_2737024
